# Transcriptome profiling of *Diachasmimorpha longicaudata* towards useful molecular tools for population management

**DOI:** 10.1186/s12864-016-2759-2

**Published:** 2016-10-12

**Authors:** M. Constanza Mannino, Máximo Rivarola, Alejandra C. Scannapieco, Sergio González, Marisa Farber, Jorge L. Cladera, Silvia B. Lanzavecchia

**Affiliations:** 1 Laboratorio de Insectos de Importancia Económica, Instituto de Genética Ewald A. Favret, Instituto Nacional de Tecnología Agropecuaria (INTA), Hurlingham, Buenos Aires Argentina; 2 Instituto de Biotecnología, Instituto Nacional de Tecnología Agropecuaria (INTA), Hurlingham, Buenos Aires Argentina; 3 Consejo Nacional de Investigaciones Científicas y Técnicas, Ciudad Autónoma de Buenos Aires, Buenos Aires, Argentina

**Keywords:** *Diachasmimorpha longicaudata*, Sex determination, Transcriptomics, Gene expression, Molecular markers

## Abstract

**Background:**

*Diachasmimorpha longicaudata* (Hymenoptera: Braconidae) is a solitary parasitoid of Tephritidae (Diptera) fruit flies of economic importance currently being mass-reared in bio-factories and successfully used worldwide. A peculiar biological aspect of Hymenoptera is its haplo-diploid life cycle, where females (diploid) develop from fertilized eggs and males (haploid) from unfertilized eggs. Diploid males were described in many species and recently evidenced in *D. longicaudata* by mean of inbreeding studies. Sex determination in this parasitoid is based on the Complementary Sex Determination (CSD) system, with alleles from at least one locus involved in early steps of this pathway. Since limited information is available about genetics of this parasitoid species, a deeper analysis on *D. longicaudata*’s genomics is required to provide molecular tools for achieving a more cost effective production under artificial rearing conditions.

**Results:**

We report here the first transcriptome analysis of male-larvae, adult females and adult males of *D. longicaudata* using 454-pyrosequencing. A total of 469766 reads were analyzed and 8483 high-quality isotigs were assembled. After functional annotation, a total of 51686 unigenes were produced, from which, 7021 isotigs and 20227 singletons had at least one BLAST hit against the NCBI non-redundant protein database. A preliminary comparison of adult female and male evidenced that 98 transcripts showed differential expression profiles, with at least a 10-fold difference. Among the functionally annotated transcripts we detected four sequences potentially involved in sex determination and three homologues to two known genes involved in the sex determination cascade. Finally, a total of 4674SimpleSequence Repeats (SSRs) were *in silico* identified and characterized.

**Conclusion:**

The information obtained here will significantly contribute to the development of *D. longicaudata* functional genomics, genetics and population-based genome studies. Thousands of new microsatellite markers were identified as toolkits for population genetics analysis. The transcriptome characterized here is the starting point to elucidate the molecular bases of the sex determination mechanism in this species.

**Electronic supplementary material:**

The online version of this article (doi:10.1186/s12864-016-2759-2) contains supplementary material, which is available to authorized users.

## Background

*Diachasmimorpha longicaudata* Ashmead (Hymenoptera: Braconidae) is a solitary endoparasitoid of larval and prepupal stages of fruit flies (Diptera: Tephritidae). It is native to Southeast Asia and has been successfully established in tropical and subtropical regions of the world. This beneficial insect is commonly used worldwide as a biological control agent in integrated pest control programs, mostly against fruit flies of the genera *Bactrocera*, *Anastrepha* and *Ceratitis* [1–5]. Many studies performed on biological parameters [6], conditions of rearing [7], and behavior [4, 8, 9] are available for this parasitoid. Inbreeding experiments provided the first description of viable diploid males in this species, and the presence of a multiple-locus CSD mechanism [10, 11] based on cytogenetic and cytological techniques and sex ratio analysis [12]. A deeper knowledge of *D. longicaudata* genomics is needed for a better understanding of the molecular basis of complex mechanisms, as the sex determination pathway.

Sex determination mechanisms can be very variable between phylogenetically close related species. The same mechanism can apparently be regulated by different genes, suggesting a rapidly evolving system [13–15]. In insects, sex determination pathways are characterized by a gene cascade that is highly variable at the top, but shares a conserved core including a common binary switch, the transformer (*tra*) gene, and at a least a common executor downstream, the *doublesex* (*dsx*) gene [15, 16]. A primary signal initiates one of the two alternative routes in the signaling cascade by leading the differential sex-specific splicing of *doublesex* (*dsx*) and *sex lethal* (*slx*) genes that control overt sexual differentiation [15]. In Hymenoptera, many models for sex determination mechanisms have been proposed, however functional studies of sex-determining genes have only been performed in few species. The most studied is the social insect species *Apis mellifera* L. (Hymenoptera: Apidae), the honeybee, which has single-locus complementary sex determination (sl-CSD) [17]. Differential sex development under this mechanism depends on the allelic composition of one sex locus. Heterozygous individuals at the *complementary sex determiner* (*csd*) locus develop as female offspring, and homozygous or hemizygous individuals develop into male offspring [18]. The heteroallelic combination of CSD activates the splicing factor *transformer* (*tra*), termed *feminizer* (*fem*), which mediates the female-specific splicing of the *dsx* pre-mRNA. In homozygous or hemizygous individuals for the *csd* locus, no functional TRA/FEM protein is present due to sex-specific splicing of the *tra* pre-mRNA, hence leading to male development [14, 18]. Other highly studied species is the gregarious parasitoid *Nasonia vitripennis* Walker (Hymenoptera: Pteromalidae), the jewel wasp. *Nasonia* has a non-CSD sex-determining system, described as the maternal effect genomic imprinting sex determination system (MEGISD). In this case, female-specific *Nvtra* (*N. vitripennis* designation for the *tra* gene) splicing depends on an autoregulatory loop, where a threshold level of maternally provided messenger is essential for female development [19].

The next generation sequencing methods (Roche 454, Solexa/Illumina, etc.) provide the opportunity for genomic exploration in non-model arthropods species wherein little or no molecular knowledge is available [20]. In particular, 454-sequencing technology based on the pyrosequencing principle has recently enabled the application of functional genomics to a broad range of arthropods [20–30].

In the current study we applied 454 sequencing technology to characterize the transcriptome of *D. longicaudata* by *de novo* assembly of three libraries (third instar male larvae, male, and female adults). Our results show the first global and big picture of *D. longicaudata* transcriptome as well as the functional annotation of its unigenes (isotigs and singletons). We also present a preliminary scenario of genes differentially expressed between sexes as a first molecular insight into the study of the molecular basis of sex determination in this non-model organism. Additionally, since no microsatellite sequences have been reported for *D. longicaudata,* we *in silico* identified and characterized SSRs with potential application as molecular markers in population genetics studies.

## Results and Discussion

### Transcriptome sequencing and assembly

To cover the transcriptome of *D. longicaudata*, third instar male larvae and adult individuals of both sexes (three pools) were used for RNA extraction. We chose adult individuals from both sexes in order to assess sex-specific expression patterns. As *D. longicaudata* is an endoparasitoid, immature stages in this species are very difficult to sample and, impossible to identify and differentiate male or female individuals in early developmental stages. A male larval sample obtained from virgin females was assessed as well in order to improve the transcriptome yield. The 454-pyrosequencing was performed independently on the three pools on a 454 GS FLX Titanium (Roche). A total of 175.7 Mbp of transcriptome data were generated, comprising 469766 raw reads with an average length of 376bp. The raw sequencing dataset was submitted to Sequence Read Archive (SRA) database, accession number SRP072867, under BioProject: PRJNA317427; samples corresponding to raw sequences of each library have been identified as NCBI BioSamples SRS1376223, SRS1376224, and SRS1376239. After filtering for adaptors, primers and low-quality sequences, 99.8 % of the first raw sequences resulted in high quality reads, representing approximately 174.3 Mbp. Low quality sequences (956 reads) were discarded.

**Fig. 1:**
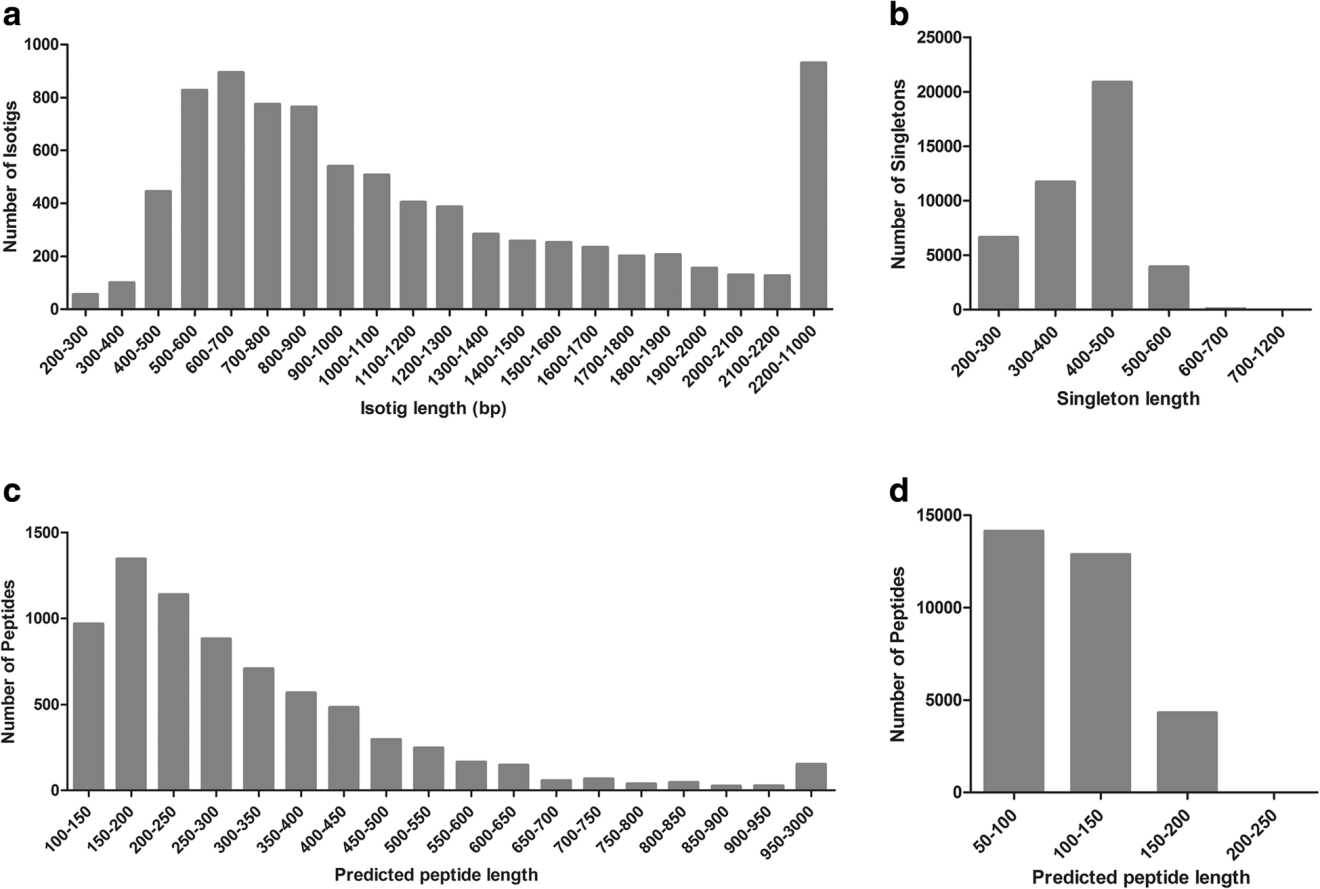
Frequency distribution of isotigs (**a**) and singletons (**b**). Sequence length and frequency distribution of predicted peptides from isotigs (**c**) and singletons (**d**). The histograms represent the number of isotigs and singletons sequences in relation to its length and the number of predicted peptides in relation to its length grouped in 50 amino acids boxes

All reads were pooled together for a *de novo* transcriptome assembly using Newbler v2.6 assembler software (Roche, IN, USA). The reads were assembled into 8483 isotigs which represent unique RNA transcripts. Isotigs which potentially are transcript isoforms generated from the same locus, are grouped together in isogroups. In our case, the assembly resulted in 7304 isogroups. Reads which did not assemble into isotigs (hereon named singletons) were clustered using CDHIT-454 algorithm to eliminate redundancy among them leaving 43203 unique singletons, summing up a total of 51686 non-redundant sequences or unigenes (Table 1). This Transcriptome Shotgun Assembly project has been deposited at GenBank under the accession GELG00000000. The version described in this paper is the first version, GELG01000000. Isotig length ranged from 200to 10956bp, with an overall average length of 1271.5bp (Fig. 1a). More than 67.2 % of the isotigs were between 560 and 1300bp long and 93.5 % of the assembled bases were incorporated into isotigs greater than 600bp. The average length of *D. longicaudata* isotigs (1271.5bp) was larger than those assembled in other non-model arthropod species [20, 21, 25]. The length distribution of the 43203 singletons ranged from 200 to 1181bp with an overall average length of 401.4bp (Fig. 1b). The length of 99.8 % of the singletons was shorter than 600bp. In an attempt to identify the possible and correct open reading frame (ORF) of all unigenes, we predicted the most probable ORF from every unigene along with its protein sequence. For this purpose, we ran the software Transdecoder [31] which resulted in 7348 ORFs in isotigs and 31337 ORFs in singletons, giving a total of 38685 best possible ORFs for all unigenes. The average predicted peptide length for isotigs was 324.5 amino acids and for singletons 109.9 amino acids (Fig. 1c and d).

**Table 1:** *D. longicaudata* transcriptome summary

Total number of qualified reads	468810
Qualified reads from larval pool	304174
Qualified reads from female adult pool	98801
Qualified reads from male adult pool	65835
Average read length (bp)	376
Total number of isotigs larger than 200bp	8483
Mean length of contig/isotigs (bp)	1271.5
Total number of singletons larger than 200bp	43203
Average length of singletons (bp)	401.4
Total number of predicted peptides	38685
Average length of peptide (amino acids)	217.2
Total of isotigs with BLASTx hits	7021
Total of singletons with BLASTx hits	20227

### Functional annotation

All 51686 unigenes were subjected to BLASTx similarity search against the NR protein database (National Center for Biotechnology Information, NCBI) followed by the BLAST2Go suite and all 38685 predicted protein sequences were run through the InterproScan full suite (to complement annotation by comparing unigenes to HMM models from PFams for example), to assign a putative function and GO terms [32, 33].

We then merged all results from InterProscan and Blast2GO by their resulting GO annotation.

Examining the BLASTx results, a7021 isotigs (82.7 %) and 20227 singletons sequences (46.8 %) had significant BLASTx matches(Table 1). The frequency of BLASTx hits from isotigs/singletons was 52.72 %, above the values previously reported for *de novo* transcriptome assemblies of related species that had no sequenced genome range from 20 to 40 %[34, 35].

The majority of matched sequences exhibited high similarity to *Apis* (11.5 %), *Camponotus* (11.4 %) and *Harpegnathor* (11 %). The BLASTx top hit distribution is showed in Additional file 1.

**Fig. 2:**
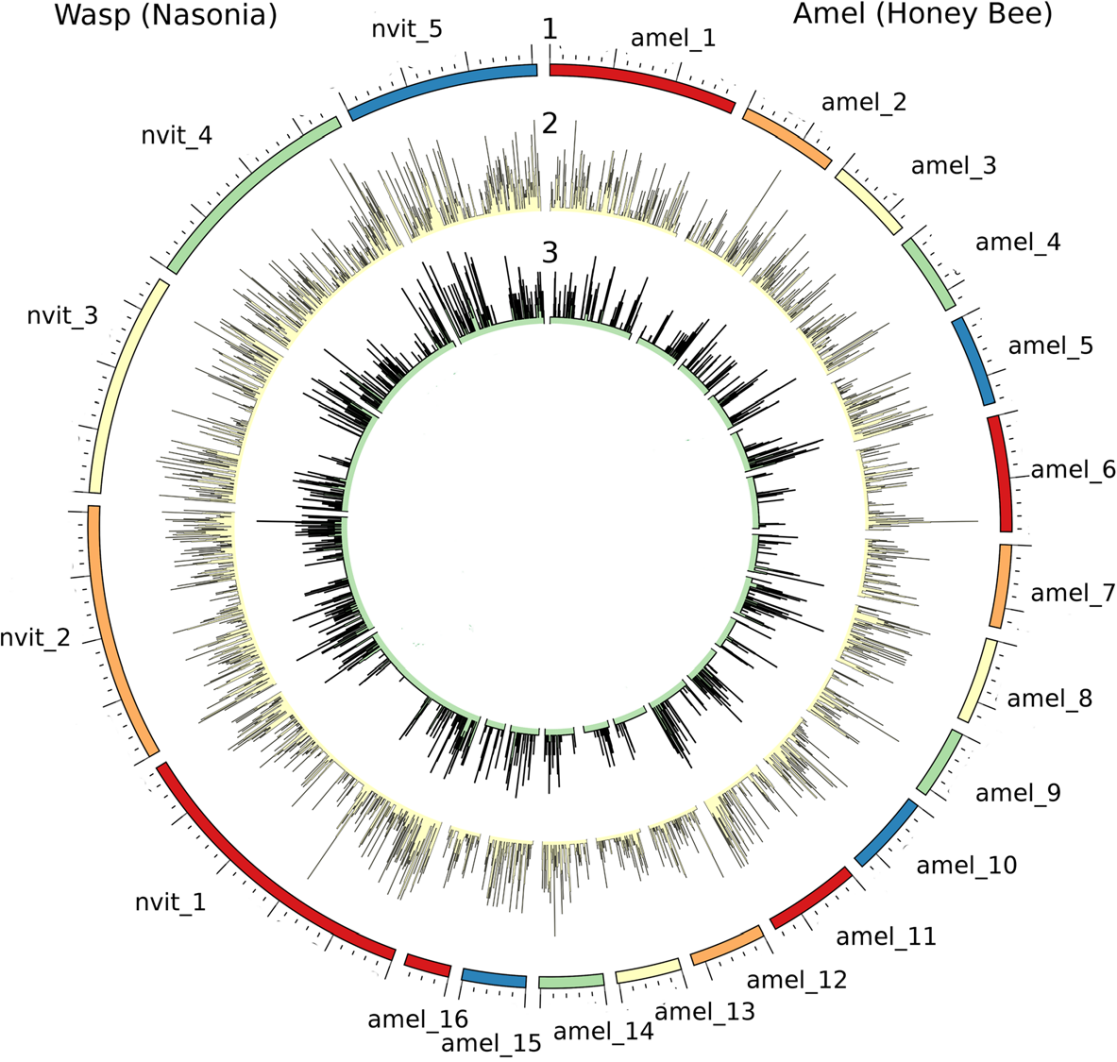
Genome and transcriptome comparisonwithrelated model species. Circular representation of: Ring 1:*N.vitripennis*(named Nvit) and *A.mellifera*(named Amel) genomes distributed in chromosomes; Ring 2: transcript density of Nvit and Amel; Ring 3: *D. longicaudata* transcript density

Genomes and transcriptomes of related model species *N. vitripennis* (Nvit) and *A. mellifera* (Amel) were used to compare and evaluate the *D. longicaudata* transcriptome distribution (Fig. 2). The uniform distribution of transcripts in this analysis reveals that our study achieved a good and unbiased representation and coverage of the *D. longicaudata* transcriptome and our data is in accordance with predicted transcript length (www.beebase.org; http://hymenopteragenome.org/nasonia/).

**Fig. 3:**
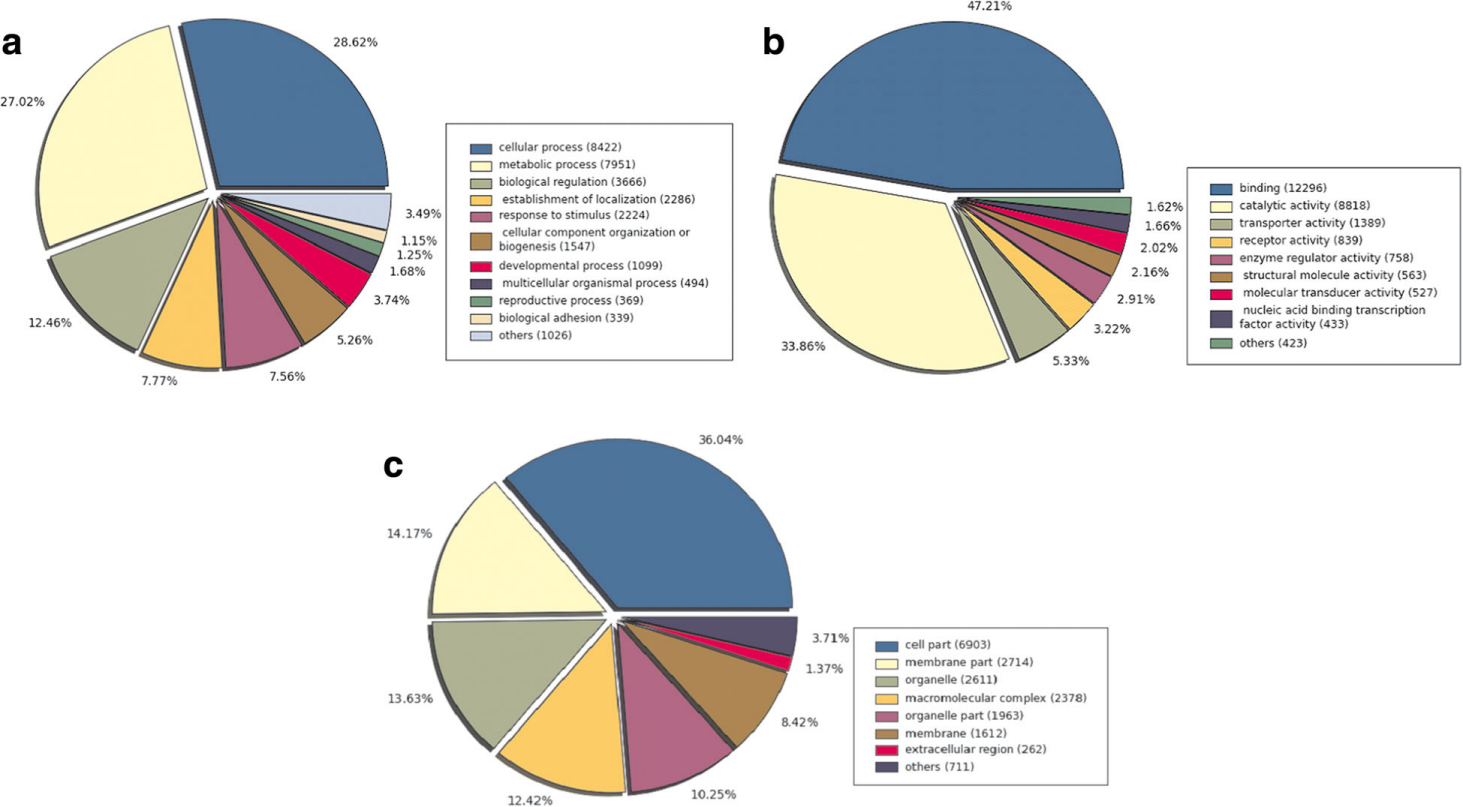
Gene Ontology (GO) assignment. The total numbers of terms annotated for each main category are 13142 for “Biological Process” (**a**), 17188 for “Molecular Function” (elemental activities) (**b**), and 9911 for “Cellular Component” (**c**)

Gene Ontology annotation: Unigenes with BLASTx hits were annotated with Blast2Go using Gene Ontology terms (GO) and Enzyme Commission categories (i.e. EC numbers). Furthermore, to complement the annotation, we ran the full InterproScan suite, which resulted in 6535 (77.0 %) and 20979 (48.6 %) significant matches for the predicted proteins translated from isotigs and singletons respectively. In summary, at least one GO term was assigned to a total of 16930 transcripts, (Fig. 3) (4483 isotigs (52.8 %) and 12447 singletons (28.8 %)).

GO terms were assigned to a total of 16930 transcripts, 4483 isotigs (52.8 %) and 12447 singletons (28.8 %). Of the GO annotated isotigs and singletons sequences, 32.65 % of terms were assigned to “Biological Processes”, 42.71 % to “Molecular functions” and 24.63 % to “Cellular components” (Fig. 3).

To further characterize *D. longicaudata*’s transcriptome, we also searched specifically for those terms associated to sex determination and related developmental processes involved in reproductionincluded in the biological processes category (Table 2). A total of four sequences were annotated as sex determination related (GO:0007530). Three of the previously mentioned sequences were annotated at a further level in the GO ontology, as primary sex determination (GO:0007538) as the other one was annotated under the term primary sex determination soma GO:0007539. There was only one sequence associated to the term primary sex determination germ-line (GO:0007542). Finally one sequence matched the female germ-line sex determination terms (GO:0018992; GO:0019099 and GO:0030237). We also detailed sequences that were homologous to those genes involved in secondary characters development. We found only 2 sequences that corresponded to femalesex differentiation processes (GO:0046660). There are seven additional sequences related to sex differentiation and related reproduction processes that were also annotated (Table 2). It is interesting to note that isotigs 00949 and 00948 are variants of the same transcript, this could represent alternative splicing for this sex associated mRNA.

**Table 2:** Sequences involved in sex determination functions and SSRs distribution

Term name	GO ID	Sequence ID	Markers and position
Sex determination	GO:0007530	HWRCMJ02HFI99; HWRCMJ02HFCXO; HWRCMJ02FPEMS; HWRCMJ02IL9LL	
Primary sex determination	GO:0007538	HWRCMJ02IL9LL; HWRCMJ02HFCXO; HWRCMJ02FPEMS	
Primary sex determination soma	GO:0007539	HWRCMJ02HFI99	SSR_801; 326–355 SSR_802: 160–171
Primary sex determination germ-line	GO:0007542	HWRCMJ02IL9LL	
Sex differentiation	GO:0007548	HWR9CMJ02FUVP3; HWR9CMJ02FK4N5;HWR9CMJ02H4520	
Germarium-derived egg chamber formation	GO:0008103	Isotig02368	SSR_537; 936–951
Sperm individualization	GO:0031011:	isotig00948	SSR_280; 1083–1102
	GO:0031011	isotig00949	SSR_279; 1083–1102 SSR_333; 2120
	GO:0008407	isotig04171	SSR_777; 592–601
Dorsal appendage formation	GO:0048814:	isotig02537	SSR_561; 1242–1251
Ovarian follicle cell differentiation	GO:0045879	isotig03388	SSR_691; 52–69 SSR_692; 1436–1450
Primary spermatocyte growth	GO:0030307	isotig03510	SSR_708; 197–206

### Comparative transcriptome analysis

**Fig. 4:**
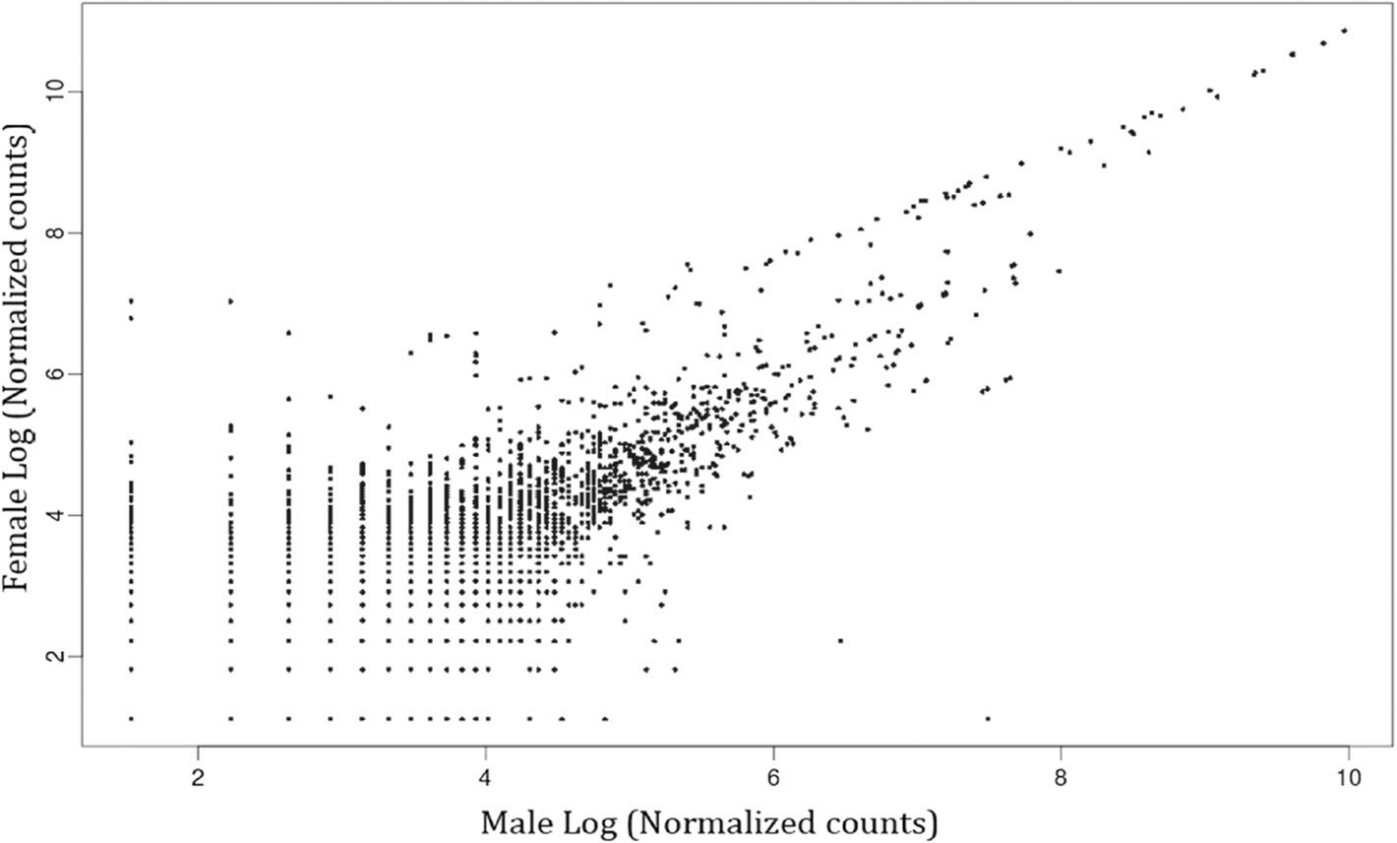
Logarithmic scale scatterplot of female/male differentially expressed transcripts. Female (y axe) and male (x axe) reads normalized to larvae of male library are represented in logarithmic scale

Adult male and female *D. longicaudata*are morphologically distinct and have different biological functions. In order to find genes involved in sex determination and differentiation pathways we compared the transcript profiles of both sexes. In our study we evaluated genes with higher differential expression pattern,likely to be involved in sex-specific functions. Sequences differentially expressed between female and male are shown in Fig. 4, evidencing that there are more sequences over-expressed in females than in males. A more detailed analysis of isotig reads numbers, considering as different those that showed at least an over 10-fold difference, revealed that there were in total 98 contigs with a tendency to be differentially expressed between the male and female adult pools. Among these sequences, 59 were overexpressed in adult females and 39 in adult males, according to RNA sequencing (Additional file 2). Among these groups of transcripts there are many isotigs that have not been annotated, suggesting that those could be part of the *D. longicaudata* specific transcriptome. It is interesting to mention that sex-specific genes show rapid sequence evolution, making its recognition difficult [36]. It would not be a surprise to find that many sex-biased genes have not yet been annotated. We could estimate, therefore, that approximately 1.2 % of the transcriptome shows at least a 10-fold change between sexes, with a majority of overexpressed transcripts in females. In addition, considering the analyzed groups and the normalization factors used trough the study, these differences might be attributed to the adult sex-specific expression patterns.

### Validation of RNA sequencing results

The next generation sequencing approach gives us a massive amount of information that must be validated in order to determine a reliable criterion for data-set comprehension. To confirm differential expression tendencies between sexesand to compare results with those obtained by RNA sequencing, we assessed a group of transcripts using RT-qPCR.

**Fig. 5:**
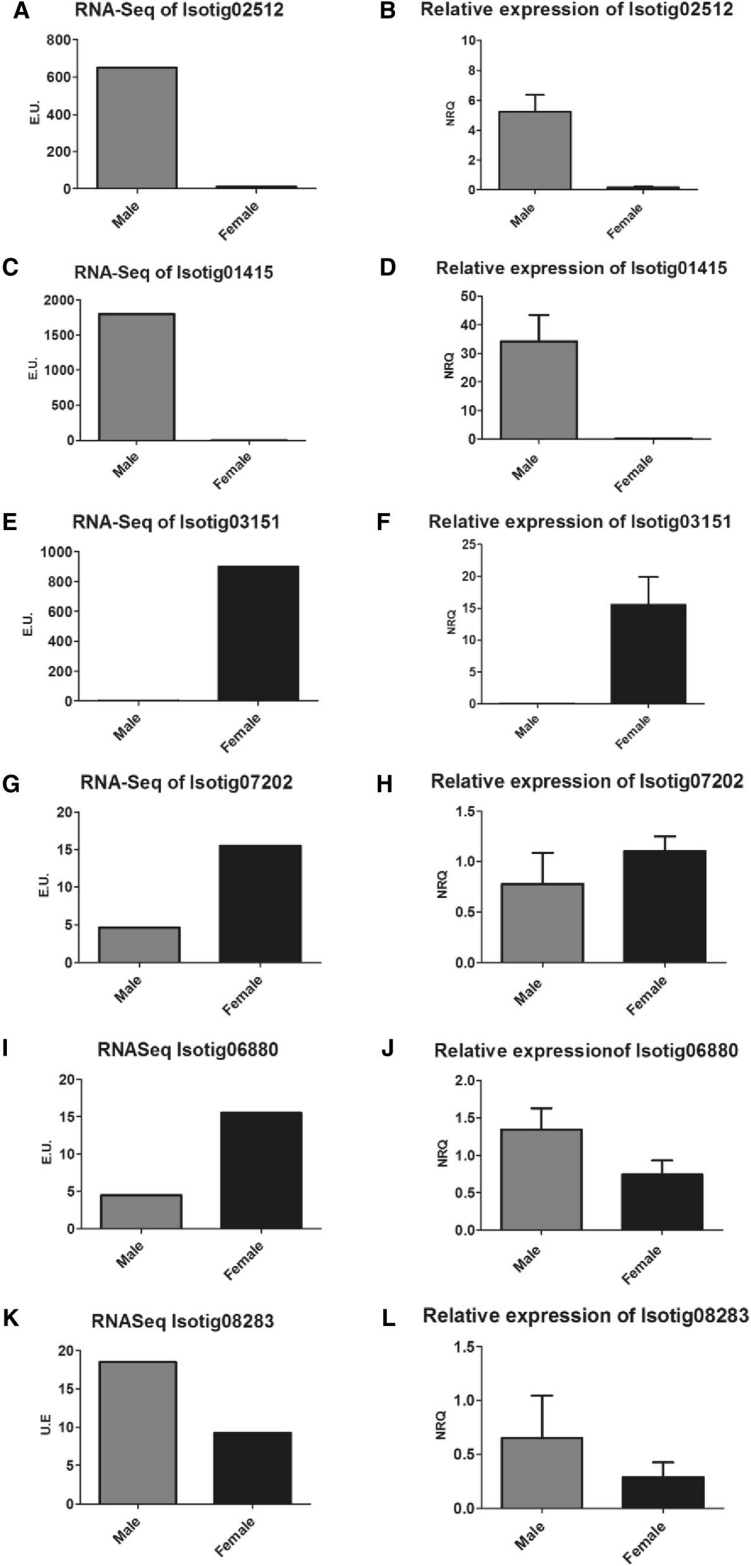
Comparative expression profiles obtained by RNA sequencing andRT-qPCR of GI. Isotigs 02512 (**a﻿/b)**, 01415 (**c/d**), 03151 (**e/f**), 07202 (**g/h**), 06880 (**i/j**), 08283 (**k/l﻿**) between male and female. EU and NRQ are Expression Units and Normalized Read Count respectively (see methods). Reference genes for RT-qPCR: *β-actin* (act) and alpha 1 *elongation factor* (α1-ef)

The comparative analysis betweenRNA sequencing and RT-qPCR of selected isotigs (named GI: genes of interest, Table 3) revealed that the RT-qPCR results were significant (*p* < 0.05), andare in concordance with the results of sequencing analysis when expression profiles of male and female were compared. A higher expression for isotig02512 was found in males using both approaches, showing around a 34-fold increasebyRNA sequencing and around 10^5^-fold increase by RT-qPCR (*p* = 5x10^−07^) (Figs. 5a and b). For isotig01415, a 329-fold increase of expression was found in malesby RNA-Sequencing, and the same pattern of expression was confirmed by RT-qPCR with a 940–fold increase (*p* = 1.5x10^−6^) (Figs. 5 c and d). On the contrary, isotig03151 presented a higher expression level in females, showing a 441-fold increase by RNA-Seq and a 15–fold increase by RT-qPCR (*p* = 10^−6^) (Figs. 5e and f). These results suggest that when analyzing normalized read count above a set threshold, the data could be reliable for assessing differential expression.

**Table 3:** Specific primers used for RT-qPCR. Gene ID and main BLAST matches

ID	Gene name	Accession no. of top BLAST hits	Sequence 5′-3′	Product size (bp)	Amplification efficiency (%)
Isotig00959	*β-actin*	[NP_511052; XP_001986647]	Fwagcacccagtcctcttgac	182	93.0
			Rvaacaccatcacccgagtcc		
Isotig00208	*α 1-elongation factor*	[BAM18878; BAG30769]	Fwtgctttcgttcccatctccg	122	90.0
			Rvtcaggcatttaccctcagcc		
isotig01415	*Odorant binding receptor*	[XP_003708550; XP_003494618]	Fwtaaggctcgcaaaggcgaat	182	93.0
			Rvtgtcgcgtgtgacgatttca		
isotig02512	*sphingomyelin phosphodiesterase*	[XM_001122062; XM_003493337]	Fwccgaggaagggtttccgaat	153	99.0
			Rvcgttcaggtcggttggtttg		
isotig03151	*Myrosinasa 1-like*	[XP_001601101; XP_001600108]	Fwaggtgcctggaatgtcagtg	205	100.3
			Rvacggtaatggcgaaacccaa		
isotig07202	*Feminization 1-like*	[NP_001153369; EFN87550]	Fwttgttccaggcagctaagca	156	103
			Rvgtcacgagtattcaggggca		
isotig06880	*Fem 1-like*	[EFN89777; EFN60633]	Fwtggttggggaggaaggaaga	239	91.3
			Rvtaaggtcacggtcgcagttg		
isotig08283	*doublesex*	[XP_003700217; ABW99103]	Fwgtcagtaccacagccagctc	152	89.7
			Rvatgttgtgggactgcggtag

### Sex determination associated transcripts

As one of the aims of this work was to detect candidate transcripts potentially involved in the sex determination mechanism of *D. longicaudata*, we looked forhomologues of genes that had already been characterized in several species as involved in sex determination [13, 17, 37, 38]. Through the sequence similarity approach (BLASTx) we searched for homologue sequences to the upstream known regulator *fem*[NP_001153369; EFN87550; NM001159897.1; XM008553448.1; XM003704454.1; XP008559611.1; EFN89777.1; XP003703392.1; XP006608847.1; EFN60633],and the known and highly conserved downstream regulator *dsx*[gi|383849166; gi|383849165; XP003395766; ABW99103.1; NP001128407.1; XP_003700217]. We found 3 sequences in the *D. longicaudata* transcript dataset that showed homology to the mentioned genes at the protein level (BLASTx): isotig07202 and isotig06880were homologues to *fem* and were identified as different transcript variants from the same locus, and isotig08283 was homologue to *dsx*.

The expression plots of RNA sequencing for both *fem* homologues show a tendency for higher expression in females (Figs. 5 g and i). However, as the normalized read count for isotig06880 was under the established cut-off for RNA sequencing (10-fold difference), the tendencies had to be confirmed by RT-qPCR.Isotig07202 higher expression in females was corroborated (*p* = 0.02) (Fig. 5 g-h).Isotig06880 showed higher expression in females by RNA sequencing and a 1.8-fold higher expression in males (*p* = 0.0003) by RT-qPCR(Fig. 5i-j). These opposite expression patterns detected for isotig06880 can be explained due to the low read counts provided by RNA sequencing. RT-qPCR has a robust statistical analysis and is, therefore, a more reliable technique. The patterns found between both *fem* variants could respond to their particular function in the different sexes. This requires further and deeper analysis and transcripts characterization.

For isotig08283, *dsx* homologue, no significant expression difference between sexes was detected (Fig. 5 k-l). The difference was lower than a 10-fold by RNA sequencing analysis and showed a *p*-value of 0.09 for RT-qPCR data analysis. This may be due to alternative splicing regulation of this transcripts rather than differential expression.

Finding genes involved in the sex determination mechanism and assessing their expression patterns in both sexes may help us have a better understanding in how and when sexual faith is determined in this non model organism. In biological control strategies, the female parasitoidis the responsible of producing the control effect, as the female lays eggs in immature stage of fruit flies species and prevent its adult emergence, thus, controlling the pest population density [1]. The knowledge about the presence of genes engaged in the sex determination cascade in this parasitoid species take a step forward towards potential sex-ratio manipulation in favor of female production which may constitute one important genetic contribution to improve the productivity and efficiency of artificial rearing of this parasitoid species. To shed light in this matter there are ongoing works on the characterization of the full transcript sequences of the sex determination genes discovered and on the analysis of their expression profiles throughout development for this species [39].

### Genetic diversity: molecular markers prediction

#### *In silico* mining of simple sequence repeats (SSR)

Using the MISA software we identified and characterized SSR (microsatellite) motifs in the *D. longicaudata* transcriptome as potential molecular markers for this species. 1233 putative SSRs within 8483isotigs and 3441 putative SSRs within the 43203 singletons were identified. The frequencies of SSR occurrence considering multiple occurrences were 15 % in Isotigs and of 8 % for singletons. For isotigs, the percentage was higher than that reported in *Spalangia endius*Walker (Hymenoptera: Pteromalidae)(8.51 %)[40] and in *Laodelphax striatellus*(Fallén) (Homoptera: Delphacidae)(7 %) [26]. A total of 982 (12 %) isotigs contained at least one SSR, and 693 SSRs (70.6 %) had sufficient flanking sequences to allow the design of appropriate unique primers. For singletons a total of 3441 contained at least one SSR, and 2082 SSRs (60.5 %) with appropriated flanking sequences characteristics to the design of unique primers.

#### Characterization of microsatellite motifs and distribution

The most frequent type of microsatellite corresponded to tetrameric (38.8 %), dimeric (24.3 %) and trimeric (14.9 %) motifs, being penta,hexanucleotideand composed repeats present at much lower frequencies (6.6 %,4.2 % and 11.1 % respectively, Additional file 3). These results are in contrast to those found in *S. endius* and *L. striatellus*, where di and trinucleotide repeats are more frequent [26, 40].

SSR motif combinations can be grouped into unique classes based on DNA base complementarities. Tetranucleotides were grouped into 27 unique classes and the numbers of unique classes possible for di and trinucleotide repeats are 4 and 10, respectively (Additional file 3). The most frequent combination for tetranucleotideswas 3 repeats of AAAT/ATTT; five repeats of AG/CT for dinucleotides and five repeats of AAT/ATT for trinucleotides (Additional file 3). These motifs are mainly AT repeats in concordance with the characterized AT-rich genomes of Hymenopteranspecies [41].

Information on the isotig and singleton identification (ID), marker ID, repeat motifs, repeat length, primer sequences, positions of forward and reverse primers, and expected fragment length are included in Additional file 3.

The microsatellite markers discovered have the potential to be used inthe characterization of the genetic variability of natural and laboratory populations of *D. longicaudata,* since little genetic information is currently available of this species [42, 43]. This parasitoid species was successfully established under artificial rearing conditions in several American countries to be used in biological control programs against Tephritid fruit flies of economic importance [44, 45]. Presently, the parasitoid is also being mass-rearedin Argentina with the same purpose [5]. The presence of established populations of this parasitoid in the wild has been reported by Schliserman et al. [46] and Oroño &Ovruski [47],originated from previous releases in the frame of biological control strategies in Argentina. The markers discovered here could be potentially useful to describe these populations at genetic level and also could be of help to develop diagnostic molecular tools to analyze the genetic quality of laboratory populations and mass rearing production of this parasitoid species (eg. inbreeding or deleterious genotypes analyses).

We analyzed the distribution of SSRs among sex determination related transcripts as a subgroup of the biological process transcripts, finding 10 potential markers associated to this subgroup. These markers are located throughout the different transcript sequences previously mentioned above (Table 2). Structure and length of terms involved and percentage of SSR found in each group are showed in Additional file 3 and SSRs distribution in sex determination associated transcripts are shown in Additional file 4. We obtained flanking sequences long enough to design primers for 4 of the 10 described SSRs. Primers, motifs and localization of these SSRs are shown in Additional file 3. Further analysis of the remaining transcript sequences by RACE PCR will increase the number of markers available for future studies on markers associated tothe sex determination pathway.

In addition, the use of microsatellite markers to construct genetic maps and to perform linkage analysis to phenotypic and behavioral traits has been explored in other insect species [48, 49] and represent a challenge to *D. longicaudata* genetics and genomics,revealing information about the regulation of main physiological pathways and understanding the genetic bases of complex traits (eg. sex determination, parasitization behavior, host allocation, host preference, etc.) all data amenable to be used for the improvement of mass-rearing and release protocols of this biological control agent [44, 45, 50].

## Conclusions

We present here the first transcriptome analysis in*D. longicaudata*. The generated transcripts dataset represents a major contribution to this non-model organism genomics and genetics, andconstitutes the first genome effort to compare the expression patterns between sexes in this parasitoid wasp. During the characterization of this transcriptome we identified two orthologues to components of sex determination pathways: *fem*and *dsx,* and at least two splicing variants of *fem*that showed differential expression between sexes. As the trigger of the sex determination cascade remains to be identified, the information provided herecould be a stepping stonefor the identification of these early signals. In addition, SSRs markers distributed throughout the transcriptome were identified, several ones associated to the sex determination group of transcripts. This work highlights the utility of transcriptome high performance sequencing as a fast and cost effective way for obtaining information on expression of species-specific genes, and for generating resources to be used in population genetic studies. The identification of genes involved in the*D. longicaudata* sex determination system together with SSR and sex determination linked SSRs discovery, are key factors for the early identification and characterization of females (responsible for fly larvae control) and its populations. This knowledge may allow performing improvements in the applied fruit fly control strategies for an effective parasitoid mass rearing toward the sex-ratio manipulation in order to increase the female production in biofactories. Finally, the possibility of identifying putative genes involved in biological processesas immunity, parasitoid-host interaction, and host preference- among others—will also provide genomic tools to improve the artificial rearing protocolsof this parasitoid, used worldwide as a biological control agent for fruit flies of economic importance.

## Methods

### Insect material

*Diachasmimorpha longicaudata* individuals used in this study came from the Instituto de Genética experimental strain (INTA Hurlingham, Buenos Aires, Argentina). This strainwas founded with individuals imported from Mexico to Tucumán (Argentina) in 1998 (SENASA, exp n° 14054/98)and introduced to our laboratory in 2001. The colony was maintained in glass flasks with water and honey and reared on third instar larvae of *Ceratitiscapitata*(Wiedemann) (Diptera: Tephritidae) as host,according to previously established protocols [6, 10].

### RNA preparation, cDNA libraries construction and 454-pyrosequencing

Total RNA was extracted using TRIzol® reagent (Invitrogen) from three pools of *D. longicaudata* individuals. Pools consisted of approximately 90mg of tissue. In each case this corresponded to 30 adult females, 30 adult males (both samples15 days after emergence), and 5 third instar male larvae (8 days after oviposition of virgin females). The larval pool was included for a more representative data set. The resultant RNA was resuspended in 50 μl of DEPC treated water. Quantity and quality of RNA were assessed using a Nanodrop (Thermo Scientific Nanodrop 2000) spectrophotometer and agarose gel electrophoresis (1 % P/V). Approximately 90μg of total RNA were processed in INDEAR (Rosario Biotechnology Institute, Rosario, Argentina) for cDNA synthesis, mRNA enrichment, libraries construction, and subsequent 454-pyrosequencing. *Diachasmimorpha longicaudata* cDNA libraries were subjected to a half plate production run on the 454-GS-FLX (Roche) sequencing instrument.

### Transcript assembly and annotation

After removing low quality sequences, filtering for adaptors and primers, all curated raw 454 read sequences from all libraries were assembled into isotigs (aligned reads from a single transcript), isotigs (alignment variants of transcripts) and isogroups (isotigs representing a genomic locus) using Newbler v2.6 Assembler software (Roche, IN, USA). Reads identified like singletons (i.e., reads not assembled into isotigs), were subjected to CD-HIT-454 clustering algorithm using a sequence identity cut-off of 90 %, which eliminates redundant sequences or artificial duplicates. BLASTx (cut-off e-value ≤ 10^−10^) searches were performed against the NCBI nr protein database in order to make an assessment of the putative identities of the sequences. Annotation and mapping were done using the software BLAST2GO, which assigns Gene Ontology terms [51] (http://www.geneontology.org), KEGG maps (Kyoto Encyclopedia of Genes and Genomes, KASS) and an enzyme classification number (EC number) using a combination of similarity searches and statistical analysis [52]. GO annotation was further completed by running the full suite of InterProScan under default parameters [33].In order to identify the most probable open reading frame (ORF) in every isotig and singleton, the program TransDecoder [31] was used with default settings. The best and most probable peptides were used as input for InterProScan. InterProScan combines different protein signature recognition methods native to the InterPro member databases into one resource that searches for the corresponding InterPro and GO annotations.

For transcriptome comparison with other model organisms the MUMMER software package, specifically Nucmer, [53] and the Circos visualization tool [54] were used. The Honey Bee Genome Sequencing Consortium assembly Amel_4.5 and Official Gene Set OGSv3.2 (www.beebase.org) and the NasoniaBase genome Assembly Nvit_1.0 (http://hymenopteragenome.org/nasonia/) databases were used.

### Differentially expressed transcripts identification (gene mining and RT-qPCR)

Total RNA was extracted from adult female and male *D. longicaudata* pools of individuals (each pool consisting of 15 adult insects of the same sex and age) using TRIzol® (Invitrogen) following the manufacturer’s protocol. The total RNA obtained was resuspended in 30 μl of DEPC treated water and quantity/quality was assessed as previously described. About 1μg of total RNA was used as template to synthesize first-strand cDNA using ImProm-II Reverse Transcriptase (Promega) and Oligo (dT) primers (Promega) following the manufacturer’s protocol. The resultant cDNA was diluted 1/10 for further use in RT-qPCR.

To identify differentially expressed contigs, reads from each library were mapped back to all the contigs which were previously assembled using all libraries together. To assess differential expression between sexes from RNA sequencing results, the relative expression level for a given transcript was calculated normalizing the read counts by the length of that transcript and divided by the number of sequence reads in the library. This value was then multiplied by the largest library sequenced. This result is then regarded as Expression Units (EU). For the identification of differentially expressed transcripts, we restricted our analysis to the transcripts expressed in all three libraries. Any isotig with less than 3 counts was discarded due to the large variability in estimating expression from low covered genes. To assess the tendencies for differential expression between male and female libraries a cut-off value of the previously mentioned quotient was set at a 10-fold difference between sexes. Female and male number of reads was normalized to larvae libraryandis represented in logarithmic scale.

We performed a Genes of Interest (GI) selection based on two criteria:1) A set of sequences which included isotigs 01415, 02512 and 03151, was selected according to significant differential expression detected trough female/male RNA sequencing resultscomparison;2) the set was selected according to sequence homology (BLASTx) that comprised three sequences isotigs 06880, 07270 and 08283. All GI were subjected to RT-qPCR analysis using IQ^TM^SYBR®GreenSupermix (Bio-Rad). Primers were designed using Primer-BLAST tool (http://blast.ncbi.nlm.nih.gov/Blast.cgi).The cycling parameters were 95 °C for 5 min followed by 40 cycles of 95 °C for 10 s and 60 °C for 45 s ending with a melting curve product amplification. Relative gene expression was analyzed by the multiple reference gene method [55]. Elongation factor 1-alpha (*ef1-α*) and *β actin* of *D. longicaudata* were used as the internal reference genes, as has been used in other insect [56, 57]. Statistical analysis was performed using the Student *t*-test.

### SSR discovery

In order to identify SSRs markers, we ran the software MISA [ref, http://pgrc.ipk-gatersleben.de/misa/misa.html].For SSR selection the criteria used werebased on the minimum number of repeats as follows: five for dinucleotide, four for trinucleotide, and three for tetra, penta and hexanucleotide motives.

To develop all pairs of primers which amplify the corresponding SSR marker in every unigene, we ran an in-house script which uses EPRIMER32 from the EMBOSS package (http://emboss.sourceforge.net/apps/cvs/emboss/apps/eprimer32.html).

## Abbreviations

CSD, Complementary Sex Determination; SSR, Simple Sequence Repeats; SNP, Single Nucleotide Polymorphism; MEGISD, Maternal Effect Genomic Imprinting Sex Determination; RT-qPCR, reverse transcription quantitative polymerase chain reaction; GI, Gene of Interest; ID, Identification; INTA, Instituto Nacional de Tecnología Agropecuaria, Argentina; SENASA, Servicio Nacional de Sanidad y Calidad Agroalimentaria, Argentina; EU, Expression Units

## Additional files

Additional file 1: Top-hit species distribution of BLASTX matches of *D. longicaudata* unigenes. Proportion of *D. longicaudata* unigenes (isotigs + singletons) with similarity to sequences from NCBI NR protein database. (TIF 45957 kb)
Additional file 2: RNA-Seq differentially expressed sequences between sexes. Dataset normalized according to sequence length and to the largest library (larvae). (DOCX 21 kb)
Additional file 3: List of SSRs and primers. (XLSX 856 kb)
Additional file 4: SSRs distribution in sex determination associated transcripts. (TIF 40828 kb)
