# Supplementary figures and images for: Transcriptome profiling of Diachasmimorpha longicaudata towards useful molecular tools for population management
Source: BMC Genomics. 2016 Oct 12;17:793. doi: 10.1186/s12864-016-2759-2 (PMC5059965; doi:10.1186/s12864-016-2759-2)

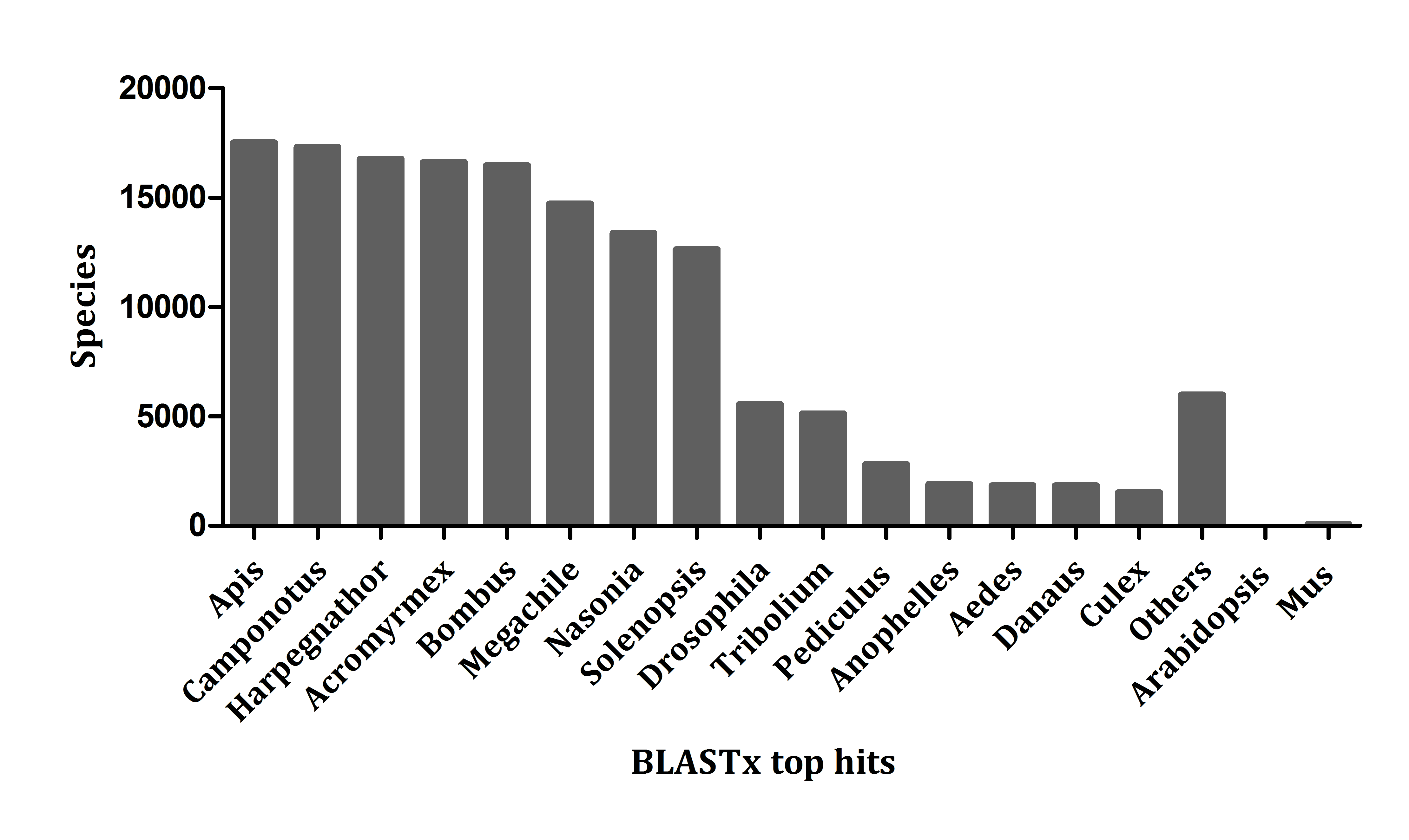

Supplement: Additional file 1: — Top-hit species distribution of BLASTX matches of D. longicaudata unigenes. Proportion of D. longicaudata unigenes (isotigs + singletons) with similarity to sequences from NCBI NR protein database. (TIF 45957 kb) [file 12864_2016_2759_MOESM1_ESM.tif]

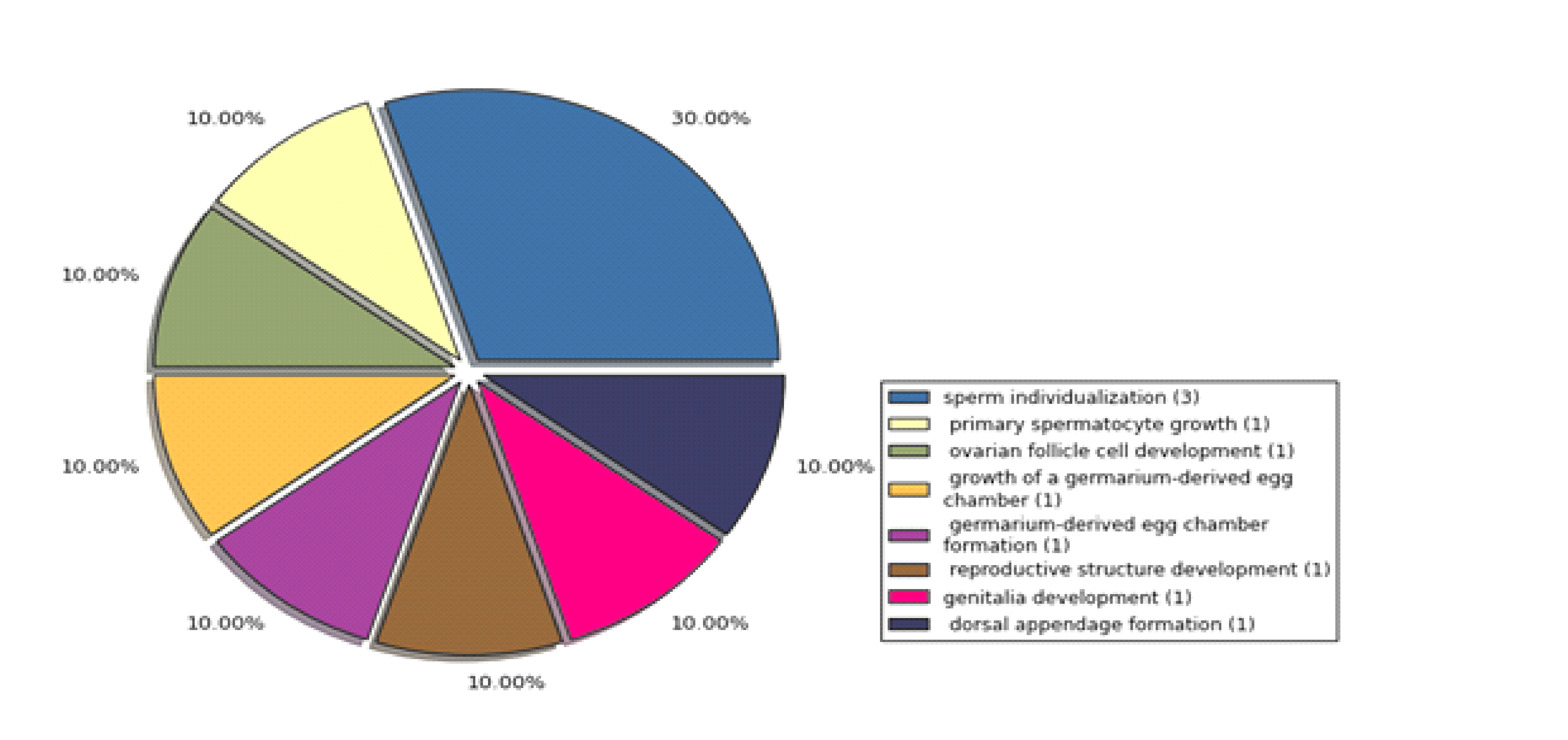

Supplement: Additional file 4: — SSRs distribution in sex determination associated transcripts. (TIF 40828 kb) [file 12864_2016_2759_MOESM4_ESM.tif]
