# Supplementary material for: Transcriptome profiling of Diachasmimorpha longicaudata towards useful molecular tools for population management
Source: BMC Genomics. 2016 Oct 12;17:793. doi: 10.1186/s12864-016-2759-2 (PMC5059965; doi:10.1186/s12864-016-2759-2)
Supplement: Additional file 2: — RNA-Seq differentially expressed sequences between sexes. Dataset normalized according to sequence length and to the largest library (larvae). (DOCX 21 kb) [file 12864_2016_2759_MOESM2_ESM.docx]

| Sequence | Male | Female | Male/Female Relativization | Female/MaleRelativization | Function (BLAST2GO) |
| --- | --- | --- | --- | --- | --- |
| isotig00184 | 37.02 | 702.62 | 0.052688509 | 18.97947056 | - |
| isotig00185 | 37.02 | 674.89 | 0.054853384 | 18.23041599 | - |
| isotig00186 | 37.02 | 656.4 | 0.056398537 | 17.73095624 | - |
| isotig00187 | 37.02 | 681.05 | 0.054357242 | 18.39681253 | - |
| isotig00188 | 37.02 | 687.21 | 0.053869996 | 18.56320908 | - |
| isotig00189 | 37.02 | 650.23 | 0.0569337 | 17.56428957 | - |
| isotig00190 | 37.02 | 702.62 | 0.052688509 | 18.97947056 | - |
| isotig00191 | 37.02 | 693.38 | 0.053390637 | 18.72987574 | - |
| isotig00192 | 37.02 | 699.54 | 0.052920491 | 18.89627229 | - |
| isotig00193 | 37.02 | 696.46 | 0.053154524 | 18.81307401 |  |
| isotig00194 | 37.02 | 684.13 | 0.054112522 | 18.4800108 | - |
| isotig00195 | 37.02 | 674.89 | 0.054853384 | 18.23041599 | - |
| isotig00298 | 32.39 | 545.46 | 0.059381073 | 16.84038283 | - |
| isotig00453 | 9.26 | 194.15 | 0.047695081 | 20.96652268 | - |
| isotig00454 | 9.26 | 194.15 | 0.047695081 | 20.96652268 | - |
| isotig00485 | 189.73 | 18.49 | 10.26122228 | 0.097454277 | Microtubule associated complex |
| isotig00486 | 175.85 | 9.25 | 19.01081081 | 0.052601649 | Metabolic process |
| isotig00574 | 4.63 | 126.35 | 0.036644242 | 27.28941685 | Acid phosphatase activity |
| isotig00583 | 4.63 | 55.47 | 0.083468542 | 11.98056156 | RNA binding |
| isotig00617 | 41.65 | 690.3 | 0.060336086 | 16.57382953 | - |
| isotig00743 | 50.9 | 523.89 | 0.0971578 | 10.29253438 | Lipid metabolic process |
| isotig00870 | 13.88 | 724.19 | 0.019166241 | 52.17507205 | - |
| isotig00946 | 9.26 | 123.27 | 0.075119656 | 13.31209503 | ATP binding |
| isotig01045 | 41.65 | 3.08 | 13.52272727 | 0.07394958 | Phospholipid binding |
| isotig01169 | 4.63 | 77.04 | 0.06009865 | 16.63930886 | Nucleic acid binding |
| isotig01186 | 97.18 | 9.25 | 10.50594595 | 0.095184194 | - |
| isotig01213 | 4.63 | 52.39 | 0.088375644 | 11.31533477 | Diacylglycerol cholinephophotransferase activity |
| isotig01288 | 4.63 | 55.47 | 0.083468542 | 11.98056156 | Zinc ion binding |
| isotig01313 | 32.39 | 3.08 | 10.51623377 | 0.095091077 | Flavin adenine dinucleotide binding |
| isotig01374 | 4.63 | 55.47 | 0.083468542 | 11.98056156 | Protein binding |
| isotig01415 | 1790.91 | 3.08 | 581.4642857 | 0.001719796 | Odorant binding |
| isotig01454 | 4.63 | 80.12 | 0.057788318 | 17.30453564 | Peptidase activity |
| isotig01516 | 4.63 | 58.55 | 0.079077711 | 12.64578834 | Acid phosphatase activity |
| isotig01998 | 9.26 | 1130.98 | 0.00818759 | 122.1360691 | - |
| isotig02013 | 23.14 | 246.53 | 0.093862816 | 10.65384615 | Hydrolase activity on ester bonds |
| isotig02092 | 4.63 | 83.21 | 0.055642351 | 17.97192225 | Protein dimerization activity |
| isotig02134 | 13.88 | 144.84 | 0.095829881 | 10.4351585 | Carbohidrate metabolic process |
| isotig02168 | 4.63 | 49.31 | 0.093895762 | 10.65010799 | COPII veisicle coat |
| isotig02245 | 13.88 | 172.57 | 0.080431129 | 12.43299712 | - |
| isotig02378 | 9.26 | 95.53 | 0.096932901 | 10.31641469 | Nucletide binding |
| isotig02397 | 4.63 | 77.04 | 0.06009865 | 16.63930886 | Protein binding |
| isotig02512 | 643.25 | 9.25 | 69.54054054 | 0.014380101 | Hydrolase activity |
| isotig02593 | 4.63 | 77.04 | 0.06009865 | 16.63930886 | Regulation of G2/M transition of mitotic cell cycle |
| isotig02652 | 185.11 | 15.41 | 12.01232966 | 0.083247799 | Transferase activity |
| isotig02713 | 4.63 | 117.1 | 0.039538856 | 25.29157667 | RNA binding |
| isotig02724 | 4.63 | 126.35 | 0.036644242 | 27.28941685 | Kinase activity |
| isotig02776 | 4.63 | 154.08 | 0.030049325 | 33.27861771 | Transferase activity |
| isotig02877 | 4.63 | 55.47 | 0.083468542 | 11.98056156 | Nucleosome, DNA bindding |
| isotig02913 | 46.28 | 3.08 | 15.02597403 | 0.066551426 | Catión transmembrane transporter activity |
| isotig02983 | 9.26 | 95.53 | 0.096932901 | 10.31641469 | Peroxisome, nucleica cid binding |
| isotig03014 | 92.55 | 3.08 | 30.0487013 | 0.033279308 | Oxidorreductase activity |
| isotig03021 | 208.24 | 9.25 | 22.51243243 | 0.0444199 | Oxidation-reduction process |
| isotig03151 | 4.63 | 1134.06 | 0.004082676 | 244.937365 | Hydrolase activity |
| isotig03162 | 9.26 | 181.82 | 0.050929491 | 19.6349892 | Polyamine biosynthetic process |
| isotig03165 | 37.02 | 3.08 | 12.01948052 | 0.083198271 | Integral to membrane |
| isotig03253 | 4.63 | 70.88 | 0.06532167 | 15.30885529 | Translation initiation facto activity |
| isotig03328 | 46.28 | 3.08 | 15.02597403 | 0.066551426 | - |
| isotig03464 | 4.63 | 117.1 | 0.039538856 | 25.29157667 | Protein binding |
| isotig03526 | 4.63 | 890.61 | 0.005198684 | 192.3563715 | Hydrolase activity |
| isotig03608 | 32.39 | 3.08 | 10.51623377 | 0.095091077 | Rab GTPase activator factor |
| isotig03671 | 32.39 | 3.08 | 10.51623377 | 0.095091077 | - |
| isotig03827 | 37.02 | 3.08 | 12.01948052 | 0.083198271 | Integral to membrane |
| isotig03861 | 32.39 | 3.08 | 10.51623377 | 0.095091077 | Transferae activity |
| isotig03986 | 46.28 | 3.08 | 15.02597403 | 0.066551426 | Piridoxal phosphate binding |
| isotig04069 | 4.63 | 61.63 | 0.07512575 | 13.31101512 | Nucleus |
| isotig04146 | 55.53 | 3.08 | 18.02922078 | 0.055465514 | Transferase activity |
| isotig04267 | 143.46 | 12.33 | 11.6350365 | 0.085947302 | - |
| isotig04351 | 41.65 | 3.08 | 13.52272727 | 0.07394958 | Spliceosomal complex |
| isotig04400 | 50.9 | 3.08 | 16.52597403 | 0.060510806 | - |
| isotig04594 | 32.39 | 3.08 | 10.51623377 | 0.095091077 | Calmodulin-dependent protein kinase activity |
| isotig04645 | 32.39 | 3.08 | 10.51623377 | 0.095091077 | - |
| isotig04715 | 87.93 | 6.16 | 14.27435065 | 0.070055726 | Catalytic activity, thiol oxidase |
| isotig04852 | 166.6 | 6.16 | 27.04545455 | 0.03697479 | - |
| isotig04873 | 4.63 | 49.31 | 0.093895762 | 10.65010799 | Clathrin adaptor complex |
| isotig04940 | 37.02 | 3.08 | 12.01948052 | 0.083198271 | Transcription, DNA-dependent |
| isotig05028 | 203.62 | 6.16 | 33.05519481 | 0.030252431 | Serine-type endopeptidase acctivity |
| isotig05190 | 78.67 | 6.16 | 12.7711039 | 0.078301767 | - |
| isotig05414 | 41.65 | 3.08 | 13.52272727 | 0.07394958 | - |
| isotig05963 | 124.95 | 3.08 | 40.56818182 | 0.02464986 | Odorant binding |
| isotig06336 | 4.63 | 55.47 | 0.083468542 | 11.98056156 | ARF GTPase activator activity |
| isotig06425 | 37.02 | 3.08 | 12.01948052 | 0.083198271 | Integral to membrane, sulfotransferase activity |
| isotig06701 | 37.02 | 3.08 | 12.01948052 | 0.083198271 | - |
| isotig06746 | 46.28 | 3.08 | 15.02597403 | 0.066551426 | - |
| isotig07165 | 4.63 | 67.8 | 0.068289086 | 14.64362851 | Transport, integral to membrane |
| isotig07236 | 4.63 | 70.88 | 0.06532167 | 15.30885529 | Receptor, phosphatidate cytidylyltransferase |
| isotig07264 | 50.9 | 718.03 | 0.070888403 | 14.10667976 | Defense response |
| isotig07719 | 41.65 | 3.08 | 13.52272727 | 0.07394958 | G-protein coupled receptor signaling pathway |
| isotig08174 | 41.65 | 3.08 | 13.52272727 | 0.07394958 | - |
| isotig08193 | 9.26 | 123.27 | 0.075119656 | 13.31209503 | Structural constituent of ribosome |
| isotig08255 | 4.63 | 70.88 | 0.06532167 | 15.30885529 | - |
| isotig08256 | 4.63 | 86.29 | 0.053656275 | 18.63714903 | Mitocondrial electron transpor, NADH to ubiquinone |
| isotig08257 | 74.04 | 6.16 | 12.01948052 | 0.083198271 | Integral to membrane, signal transduction |
| isotig08345 | 74.04 | 3.08 | 24.03896104 | 0.041599136 | - |
| isotig08350 | 32.39 | 3.08 | 10.51623377 | 0.095091077 | - |
| isotig08359 | 50.9 | 542.38 | 0.093845643 | 10.65579568 | - |
| isotig08433 | 13.88 | 283.51 | 0.048957709 | 20.42579251 | ATP-binding; protein serine/threonine kinase activity |
| isotig08435 | 18.51 | 292.76 | 0.063225851 | 15.81631551 | - |
| isotig08441 | 129.57 | 1426.82 | 0.090810333 | 11.01196265 | - |
